# First detection and molecular characterization of rabbit hemorrhagic disease virus (RHDV) in Algeria

**DOI:** 10.3389/fvets.2023.1235123

**Published:** 2023-08-31

**Authors:** Lynda Sahraoui, Hichem Lahouassa, Samia Maziz-Bettahar, Ana M. Lopes, Tereza Almeida, Hacina Ainbaziz, Joana Abrantes

**Affiliations:** ^1^Laboratory of Animal Health and Production, Higher National Veterinary School of Algiers, Algiers, Algeria; ^2^Institute of Veterinary Sciences, Saad Dahlab University of Blida1, Blida, Algeria; ^3^CIBIO, Centro de Investigação em Biodiversidade e Recursos Genéticos, InBIO Laboratório Associado, Campus de Vairão, Universidade do Porto, Vairão, Portugal; ^4^BIOPOLIS Program in Genomics, Biodiversity and Land Planning, CIBIO, Vairão, Portugal; ^5^UMIB - Unit for Multidisciplinary Research in Biomedicine, ICBAS - School of Medicine and Biomedical Sciences, University of Porto, Porto, Portugal; ^6^ITR - Laboratory for Integrative and Translational Research in Population Health, Porto, Portugal; ^7^Departamento de Biologia, Faculdade de Ciências da Universidade do Porto, Porto, Portugal

**Keywords:** Algeria, rabbits, rabbit hemorrhagic disease virus, epidemiology, RHDV2, GI.2

## Abstract

Since the first detection of rabbit hemorrhagic disease (RHD), the rabbit hemorrhagic disease virus (RHDV) has been responsible for high morbidity and mortality worldwide, both in domestic and in wild rabbits. Despite the apparent control of RHD in rabbitries through vaccination, several studies highlighted the rapid evolution of RHDV by recombination, which may facilitate the emergence of new pathogenic strains. The aim of this study was to confirm the presence and characterize RHDV in Algeria. For this, rabbit samples were collected in the north of Algeria, between 2018 and 2021, from small farms where the virus was suspected after the sudden death of a high number of rabbits, and from healthy hunted wild rabbits. The domestic rabbits revealed clinical signs and lesions that were suggestive of RHD. RT-PCR showed that 79.31% of the domestic rabbit samples were positive for RHDV, while in 20.69%, including the hunted rabbits, the virus was not detected. Phylogenetic analysis of the Algerian strains allowed the confirmation and identification as GI.2 (RHDV2), and showed a close relation to GI.3P-GI.2 recombinant strains, suggesting a potential introduction from other countries, with an older strain potentially originated from neighboring Tunisia, while more recent isolates grouped with strains from North America. Our study reports for the first time the presence of GI.2 (RHDV2) in Algeria with multiple routes of introduction. Consequently, we propose that RHDV control in Algeria should be based on epidemiological surveys in association with an adequate prophylactic program.

## Introduction

Rabbit hemorrhagic disease (RHD) is a highly contagious and fatal disease, and therefore a serious threat to the rabbit production industry and wild rabbit populations. RHD-affected animals show histopathological lesions in the liver, lungs, spleen, heart and kidneys, associated with disseminated intravascular coagulation and acute hepatitis (1).

The RHD virus (RHDV) is the etiological agent of this disease, and belongs to the genus *Lagovirus*, family *Caliciviridae* (2). According to (3), this genus encompasses a single species, *Lagovirus europaeus*, separated in two genogroups, GI (rabbit lagoviruses) and GII (hare lagoviruses), which circulate in leporid populations. The GI genogroup is further subdivided into the pathogenic genotypes GI.1 (classical RHDV), with variants GI.1a to GI.1d, and GI.2 (RHDV2/b), and the non-pathogenic genotypes GI.3 (rabbit calicivirus, RCV-E1) and GI.4 (RCV-A1 and RCV-E2) (3).

The growing number of complete RHDV genome sequences allows detailed analyses of the genetic diversity. Indeed, several recombinant strains have been discovered, suggesting that recombination leads to a rapid evolution of the virus and the emergence of novel pathogenic strains (4–6). These strains often combine non-structural and structural genes of different genotypes, e.g., GI.3P-GI.1d, GI.1bP-GI.2, GI.3P-GI.2, and GI.4P-GI.2 (4, 5, 7–11). In addition, triple recombinants have been described, composed by the p16 of GI.4, the remaining non-structural genes of either GI.1b or GI.3, and the structural genes of GI.2 (10).

The first outbreak of RHD, caused by RHDV GI.1c, was described in China in 1984 in a group of rabbits imported from Germany (12). Thereupon, the virus quickly spread worldwide. It was first detected in Europe (Italy) in 1986 (13), in the Americas (Mexico) in 1988 (14) and in Africa (Egypt) in 1988 (15). RHDV GI.2 was detected for the first time in France in the summer of 2010 (16) and then dispersed throughout the world (17–22). Unlike GI.1, GI.2 is able to cause death in kittens (<2 months old) (23). Furthermore, the host range of RHDV GI.1 is almost exclusively limited to European rabbits (*Oryctolagus cuniculus*), while RHDV GI.2 is known to affect different species of hares, cottontails and jackrabbits (22, 24–29). GI.2 RNA has also been found in non-leporid species (30, 31).

In Algeria, the production of rabbit meat is estimated at 8,250 tons/year, ranking 10th worldwide, which represents 0.7% of the global production (32). However, as an African country, Algeria lags behind Egypt, which ranks sixth with 48,000 tons/year (32). The annual consumption of rabbit meat is very low, with 0.36 kg/capita (32). Indeed, rabbit farming in Algeria has a weak importance due to the absence of structure of the sector, unlike the other meat sectors such as ovine, bovine and poultry. Algerian domestic rabbits are represented by a local population, raised in the center north of the country, where vaccination against RHD and/or myxomatosis is not systematically applied in rabbit breeding and, if it is performed, it does not follow a regular program (Ain-Baziz, personal communication). In parallel to domestic rabbits, wild rabbits are hunted and consumed, but they had not yet been properly studied and described.

Recently, outbreaks of RHD have been recorded in countries in North Africa, including Morocco (8), Tunisia (33, 34) and Egypt (35–37). In addition, RHD was strongly suspected in Algeria due to the observation of high mortality rates and clinical signs in domestic rabbits, suggestive of the presence of the virus since at least 2018, but has never been confirmed. This study describes the identification and characterization of RHDV for the first time in Algeria.

## Materials and methods

### Sample collection and examination

**Figure 1:**
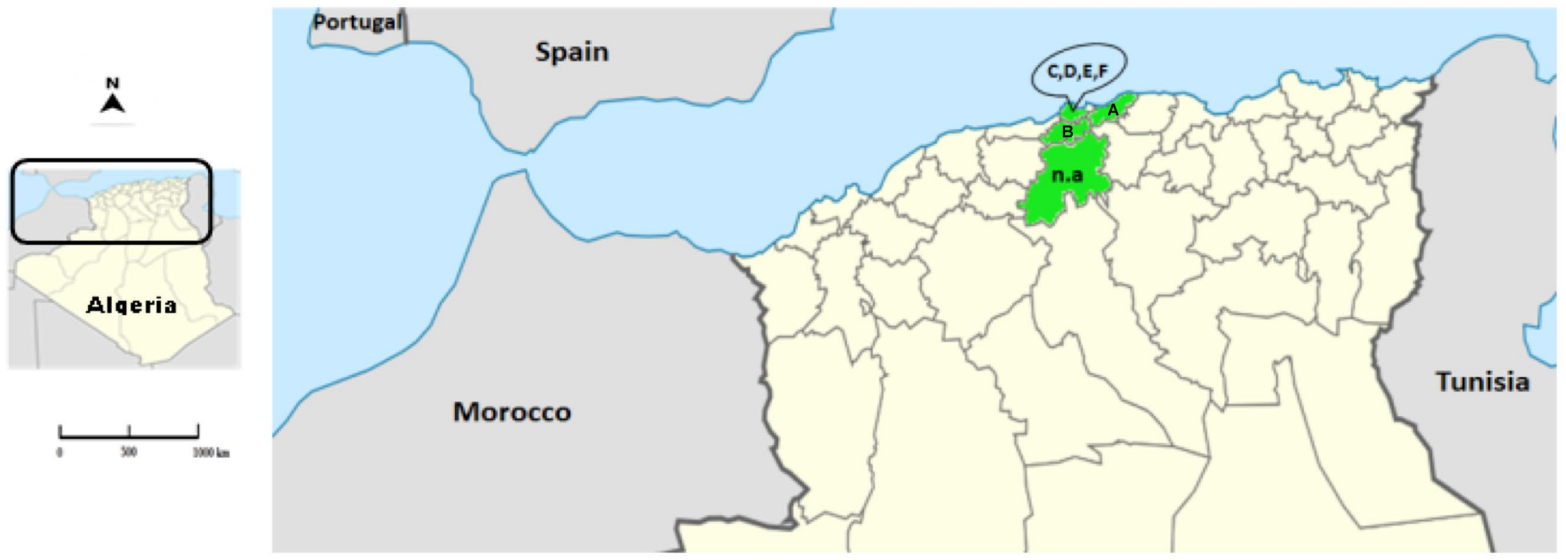
Location of the Algerian RHD-suspected cases. The map represents the location of the collected samples (provinces appear in green) in the north of Algeria. The letters correspond to the location of the farms (A: Blida; B: Boumerdes; C–F: Algiers). The abbreviation n.a. (not applicable) represents the samples of wild animals from Medea.

Rabbit samples were collected between 2018 and 2021 from domestic and wild rabbits in four provinces of the northern central region of Algeria: Algiers, Blida, Boumerdes and Medea (Figure 1; Table 1).

**Table 1:** Information of the rabbit samples collected.

Rabbit farm	Date of sampling	Locality	Number of samples	Age (days)	Sex	Sample code
A	2018	Blida (Soumaa)	2	30	M	001, 002
B	2019	Boumerdes (Thénia)	3	53 to 56	M	003–005
C	December 2020/March 2021	Algiers (Oued Smar)	10	45 to 120	9M/1F	022–027, 079–082
D*	March 2021	Algiers (El-Harrach)	11	25 to 180	9M/2F	052–056, 067–071, 076
E	March 2021	Algiers (Eucalyptus)	2	28 and 30	F	083, 084
F	March 2021	Algiers (Baraki)	1	90	F	085
n.a.	October and November 2020	Medea	4	n.d.	n.d.	006, 007, 010, 011

n.a.: not applicable, n.d.: not determined, M: male; F: female.

* Pet rabbit farm.

### Virological analysis

Liver samples were collected from RHD-suspected domestic rabbits, both male and female, aged between 25 and 120 days (*n* = 29). The animals originated from six rural farms where vaccination programs against RHDV were not applied. In addition, liver samples from apparently healthy wild rabbits (*n* = 4) were obtained from hunters during October and November 2020. All samples were collected aseptically, placed individually in sterile bags and stored at −20°C until further processing.

### Clinical and histopathological examination

In the RHD-suspected cases, the clinical diagnosis was based on the clinical history obtained from farmers and veterinarians. Necropsies were performed at the farms or at the laboratory of the veterinary school (ENSV, Algeria). In the domestic animals, three were not suspected as RHDV-infected, while 26 presented suspicious carcasses. From these, samples of liver, lung, thymus, trachea, kidney and spleen were collected for histopathological examination. Tissue samples were fixed in 10% buffered formalin and embedded in paraffin. Sections of 3 μm thick were stained with hematoxylin and eosin for routine microscopical examination.

### Molecular analysis

Liver samples (*n* = 33) were sent to CIBIO-InBIO/UP, Portugal, where RNA extraction, genome amplification and sequencing was performed. Total RNA was extracted using the GeneJET RNA Purification kit (Thermo Fisher Scientific) and reverse transcribed with the NZY First-Strand cDNA synthesis kit (Nzytech) according to the manufacturer’s protocol. RHDV presence was confirmed with two pairs of primers: RHDV4831F + EBHSV_VP60_0467R, which amplify a fragment of ~900 bp and that includes the recombination site between RdRp/VP60, and RHDV6186F + RHDV6748R, which amplify a fragment of 563 bp of the capsid gene (38). For a subset of the samples (*n* = 7) chosen according to their date of sampling, the remaining sequence of the capsid gene was PCR-amplified using the methodology described in (4) (primers and PCR conditions available from the authors upon request). All the PCRs were performed with 1 μl of the cDNA reaction in a final volume of 10 μl containing 5 μl of Phusion Flash High-Fidelity PCR Master Mix (Thermo Fisher Scientific) and 2 pmol of each oligonucleotide. Positive PCR products were purified and sequenced on an automatic sequencer (ABI PRISM 3130xl Genetic Analyzer, PE Applied Biosystems) using the amplification primers.

### Phylogenetic analysis

The sequences of the Algerian strains were aligned with lagovirus sequences retrieved from GenBank^1^ in BioEdit version 7.2 (39). Phylogenetic analyses were conducted using MEGA 11 (40). Maximum-likelihood phylogenetic trees were inferred for the full length VP60 gene and for the partial fragment of RdRp. For both ML trees, the GTR + G + Γ4 model of nucleotide substitution was used, as determined in the same software and according to the lowest AICc value, and branch support was obtained from 1,000 bootstrap replicates. The partial deletion option (95%) was used to handle missing data.

## Results

### Clinical and *post mortem* examination of RHD-suspected rabbits

RHD-suspected domestic rabbits (*n* = 29) were subjected to evaluation of clinical signs and *post mortem* examination (see Supplementary Figure 1). The affected rabbits revealed anorexia, apathy, prostration and breathing difficulties. In weaned rabbits, neurological signs were observed, with a few cases of epistaxis and anal bleeding in adults.

*Post mortem* examination of the carcasses revealed RHD-compatible alterations, with lesions affecting a large number of organs (data not shown). Indeed, all carcasses, except those from rabbits 003, 004, and 005, showed enlargement and hemorrhages of the lungs, heart and thymus. The kidneys and spleen were congested and enlarged. Petechial hemorrhages were seen on the surface of the caeca. The tracheas were hyperemic and tracheal mucosa contained frothy fluid. In addition, icteric discoloration of the visceral mucosa was observed and disseminated intravascular coagulation (DIC) was observed in the majority of the cases. The liver appeared pale and with reduced consistency. Histological examination also revealed pathological alterations in the tissues. The liver showed degenerative lesions in hepatocytes and multi-focal necrosis. Signs of degeneration and necrosis were also observed in the kidneys, spleen and heart. In the lungs and trachea, signs of hemorrhage and edema were noted.

### RHDV detection and preliminary genetic characterization

Clinical and histopathological examinations were compatible with RHD. Subsequently, molecular analysis was performed to confirm the presence of RHDV in liver samples. RT-PCRs showed that 23 out of 29 (79.31%) samples were positive for RHDV, while in 6 (20.69%) the virus was not detected. The four hunted wild rabbits were among the negative samples.

Combination of VP60 sequencing results (*n* = 7; Algeria 002, 024, 027, 083, 076, 068, and 053; GenBank accession numbers OR296430-OR296436, respectively) and nucleotide BLAST analysis^2^ allowed to characterize all these RHD positive cases as infected by GI.2.

### Phylogenetic analysis

**Figure 2:**
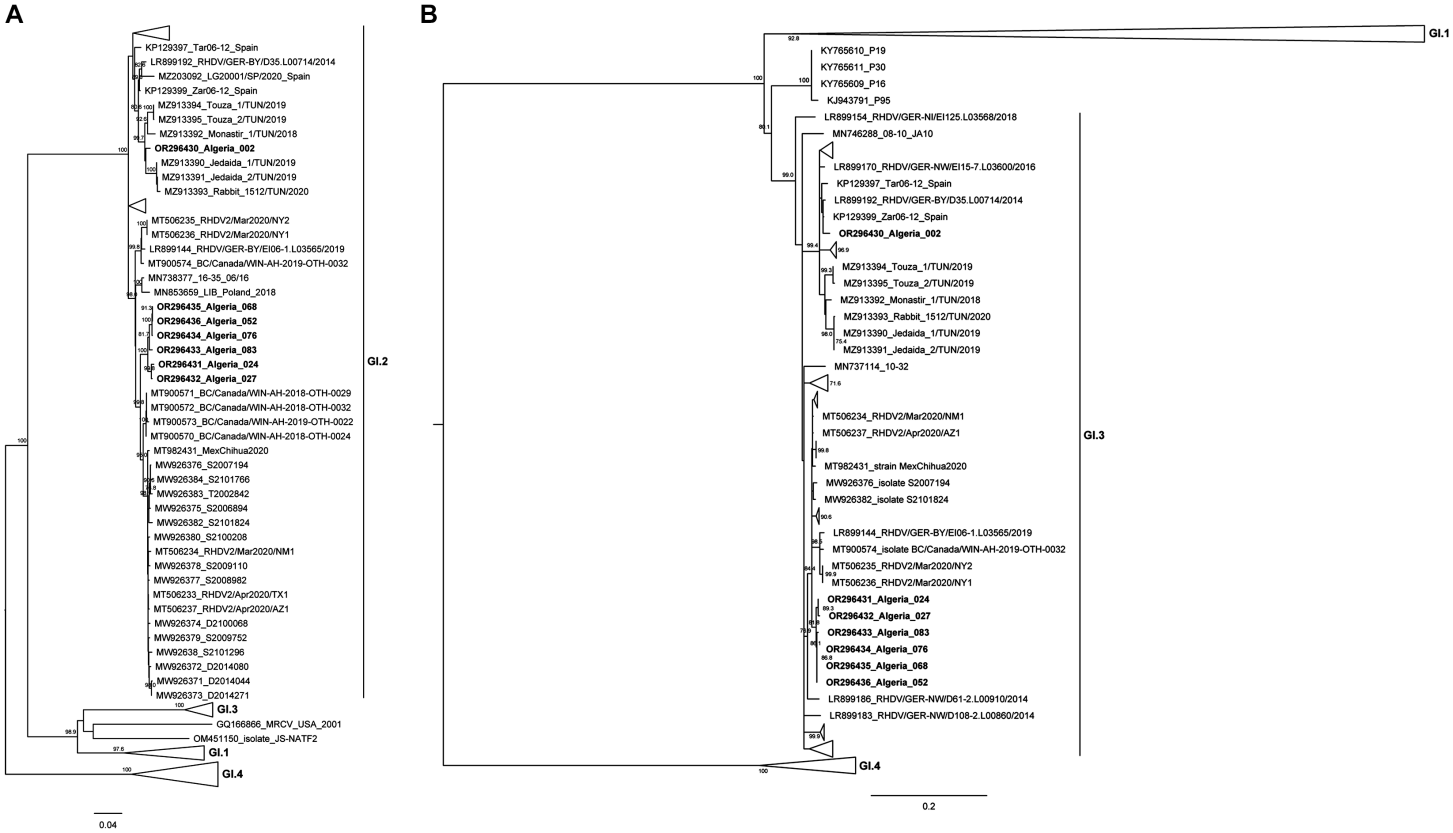
Maximum-likelihood (ML) phylogenetic analyses. **(A)** ML tree for full-length sequences of the VP60 gene (*n* = 758 sequences; nucleotide positions 5,296–7,038; nucleotide substitution model GTR + G + Γ4). **(B)** ML tree for partial sequences of RdRp (*n* = 758 sequences; nucleotide positions 4,621–5,295; nucleotide substitution model GTR + G + Γ4). For better visualization, groups are collapsed. Horizontal branch lengths are drawn to scale of nucleotide substitutions per site and the trees are mid-point rooted. The percentage of trees in which the associated taxa clustered together was determined from 1,000 bootstrap replicates and is shown next to the branches (only bootstrap values ≥70 are shown).

In order to further establish the evolutionary relationships of the Algerian strains with the available pathogenic and non-pathogenic lagovirus strains, and, particularly, with recent African isolates, ML phylogenetic trees were constructed with full coding sequences of the VP60 gene (Figure 2A) and with partial sequences of the non-structural RdRp (Figure 2B).

Analysis of the VP60 ML tree revealed that all Algerian strains clustered within the strongly supported GI.2 group (bootstrap value 100), but they were further distributed into two distinct clusters. Indeed, the strain Algeria 002 grouped with the Tunisian GI.2 strains isolated from 2018 to 2020 (bootstrap value of 99) (33). The remaining six strains isolated between 2020 and 2021 (Algeria 024, 027, 052, 068, 076, and 083) appeared closely related to North American GI.2 strains (from 2020 to 2021; bootstrap value of 99.8). The ML tree for the partial sequence of RdRp (Figure 2B) showed that the Algerian strains clustered within a strongly supported group (bootstrap value 99) composed of known GI.3 strains. Combination of the results of both trees indicates that all Algerian strains are GI.3P-GI.2 recombinants. Nucleotide Blast analyses are in line with the results from the ML trees (see Supplementary Tables 1, 2). Indeed, for the VP60 and VP10 genes and the partial RdRp sequence, the Algerian strain 002 was more similar to European strains collected between 2011 and 2016 and Tunisian strains from 2018 to 2020 (nucleotide identity: 96.48–98.38%), suggesting an European origin. For the Algerian strains 052, 068, 076, 083, 027, and 024, both the VP60 and VP10 sequences and the partial RdRp sequence were more similar to strains from the USA, Mexico and Canada collected from 2018 onwards (nucleotide identity 98.67–97.15%). For these more recent Algerian strains, there seems to be a link with North American countries.

The spatio-temporal distribution of Algerian strains seems to match their clustering in the VP60 phylogenetic tree. Indeed, Algeria 002 was collected in Blida in 2018, thereby representing the older strain of this study. The Algerian 024, 027, 052, 068, 076, and 083 strains belong to the same cluster and they can be further subdivided into two well-supported sub-clusters: the first includes Algerian strains 024 and 027 that were collected in Algiers-Oued-Smar in 2020, while the second contains Algerian strains 052, 068, 076 and 083 that were sampled in 2021 from a pet rabbit farm in Algiers-El Harrach.

## Discussion

RHD is among the diseases with the highest negative impact in wild and domestic rabbits. In rabbitries, control of RHD is achieved through adequate vaccination protocols, which should be tailored to the epidemiological situation. In order to better understand and control RHD in Algeria, we conducted a study to characterize the diversity of RHDV strains circulating in Algerian domestic and wild rabbits.

Based on the clinical signs, lesions and epidemiology found in the rabbits sampled, which are coherent to those described previously for RHD in other countries (41), this seems to be the first report of RHD in Algeria. Indeed, similar signs were observed in our study, including sudden death, high mortality, epistaxis, jaundice, and respiratory and neurological signs. These clinical alterations are due to the high pathogenicity of RHDV. Both GI.1 and GI.2 induce similar symptoms; yet, the latter induces a more prolonged disease when compared to GI.1 (42). While the observed macroscopic lesions were variable, lungs and liver were consistently the most affected organs, in agreement with earlier descriptions (4, 23). In addition, icterus jaundice was present in several carcasses. The reported alterations were the consequence of multiple organ failure resulting from lung edema and hemorrhages, adrenocortical necrosis, circulatory disorder of the kidneys and hepatic necrosis (43). There was a good agreement between the results of the clinical examination and the molecular characterization, with 26 and 23 samples identified as RHD and RHDV-positive, respectively. The difference in the results might be attributed to sample degradation due to poor preservation or the presence of low viral loads that hampered viral RNA detection.

Our study describes the first confirmed cases of GI.2 in Algeria. Since its detection in France in 2010, GI.2 rapidly spread worldwide (5, 6, 19, 44) and seems to have replaced GI.1 (19). In North Africa, GI.2 has been found in domestic and wild rabbits. Indeed, the virus was reported at variable detection rates ranging from 32% in Egypt to over 87% in Morocco and 96% in Tunisia (*n* = 50, 57, and 24, respectively) (8, 34, 35). The detection of GI.2 in our samples seems to be associated with a high mortality rate (approximately 80%, as reported by veterinarians and farmers). In agreement with our findings, it has been reported that GI.2 strains induced, at least, 80% mortality (45). Moreover, in this study, RHDV positive samples were collected from rabbits at different ages and sexes, thereby contributing to confirm that GI.2 infects male and female rabbits at all ages (5, 6, 8, 19, 33, 46).

Regarding the evolutionary relationships of the Algerian strains, inclusion of sequences from other African (8, 33, 34), American and European countries in our phylogenetic analyses (5) potentially elucidated the origin of the Algerian strains. Indeed, Algerian strains seem to have two distinct origins, resulting from independent introductions. Strain Algeria 002 possibly originated from Tunisia, while the remaining Algerian strains are more related to North American strains. This is in line with the multiple routes of introduction of GI.2 in Africa already suggested by other studies, but contrasts with previous findings in other GI.3P-GI.2 strains as they were derived from a single introduction with a likely European origin (33). The putative role of North American countries in the spread of GI.2 into Algeria is possibly associated with rabbit commercial routes. Canada and Algeria have a strong commercial relationship, with Algeria ranking as the second largest market of Canada.^3^ The other African countries where GI.2 has been reported, such as Morocco (8), Ghana and Nigeria (47), had no role in the epidemiology of the disease in Algeria as their strains were not closely related to the Algerian strains.

During the last decade, the rapid expansion of the rabbit industry in Algeria, associated with an increased popularity of rabbits as pets, in particular the breed of foreign rabbits, might have contributed to the incursion of GI.2 in Algeria. Recent findings suggest that, after a GI.2 infection, surviving rabbits can act as virus carriers for several weeks (48). Thus, importation of apparently healthy rabbits might be a source of RHDV GI.2 to Algeria and, therefore, should be highly regulated.

In conclusion, our study detected and characterized, for the first time, RHDV, in particular GI.2, in Algerian rabbits. The Algerian GI.2 strains seem to have distinct origins, possibly linked with rabbit trade. The detection of GI.2 in Algeria, with different origins and within a relatively short time, highlights the existence of multiple routes of GI.2 introduction and reinforces the importance of implementing intensive epidemiological surveillance and a national prophylactic program tailored against circulating RHDV strains.

## Data availability statement

Data produced in this study is available at GenBank (https://www.ncbi.nlm.nih.gov/genbank/) under accession numbers OR296430-OR296436.

## Ethics statement

The animal study was approved by Direction Générale des Forêts, Ministère de l’Agriculture et du Développement Rural, République Algérienne Démocratique et Populaire (N4529/BOG/DPFF/DGF-20) for the samples from the wild animals. Ethical review and approval was not required for the domestic animals because samples for laboratory diagnostic were obtained from deceased animals. The study was conducted in accordance with the local legislation and institutional requirements.

## Author contributions

LS and SM-B performed the necropsies, collected the samples, and characterized the macroscopic and microscopic lesions. JA, AL, and TA performed the virological analysis and revised the manuscript. LS, HL, SM-B, AL, JA, and TA analyzed the data. LS, HL, SM-B, and HA-B wrote the manuscript. All authors contributed to the article and approved the submitted version.

## Funding

This work is a result of the project LAGMED (www.lagmed.eu) supported by Fundação para a Ciência e Tecnologia, FCT Portugal (PRIMA/0003/2018) and General Directorate for Scientific Research and Technological Development (DGRSDT) in Algeria, PRIMA programme, an Art. 185 initiative supported and funded under Horizon 2020, the European Union’s Framework Programme for Research and Innovation. FCT also supported the Junior Researcher grant of AL (CEECIND/01388/2017) and the Assistant Researcher grant of JA (CEECIND/00078/2017).

## Conflict of interest

The authors declare that the research was conducted in the absence of any commercial or financial relationships that could be construed as a potential conflict of interest.

## Publisher’s note

All claims expressed in this article are solely those of the authors and do not necessarily represent those of their affiliated organizations, or those of the publisher, the editors and the reviewers. Any product that may be evaluated in this article, or claim that may be made by its manufacturer, is not guaranteed or endorsed by the publisher.

## Footnotes

^1^https://www.ncbi.nlm.nih.gov/genbank

^2^https://blast.ncbi.nlm.nih.gov/Blast.cgi

^3^https://www.international.gc.ca/country-pays/algeria-algerie/relations.aspx?lang=eng
