# Supplementary material for: First detection and molecular characterization of rabbit hemorrhagic disease virus (RHDV) in Algeria
Source: Front Vet Sci. 2023 Aug 31;10:1235123. doi: 10.3389/fvets.2023.1235123 (PMC10513046; doi:10.3389/fvets.2023.1235123)
Supplement: Supplementary file 1 [file Data_Sheet_1.PDF]

Supplementary Material

First detection and molecular characterization of rabbit hemorrhagic disease virus (RHDV) in Algeria

Lynda Sahraoui, Hichem Lahouassa, Samia Maziz-Bettahar, Ana M. Lopes, Tereza Almeida, Hacina Ain-Baziz, Joana Abrantes*

*** Correspondence:** Joana Abrantes: jabrantes@cibio.up.pt

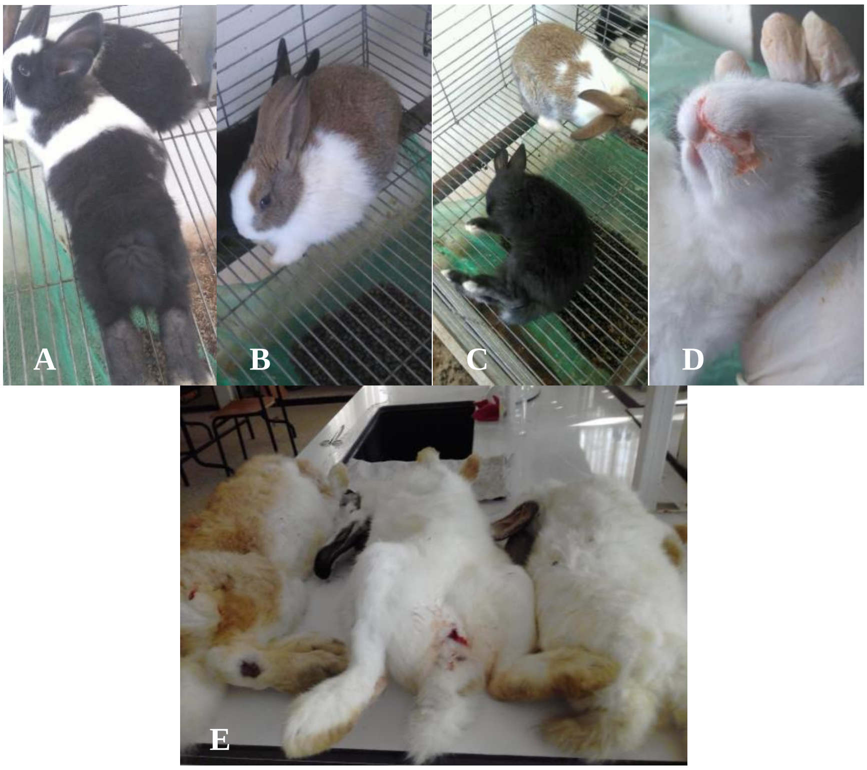

Supplementary Figure 1. Clinical signs found in RHD-suspected domestic rabbits. A. Analgesic position of the rabbit due to respiratory difficulties. B. Rabbit with neurological signs before death. C. Prostrated rabbit in the corner of the cage. D. Affected rabbit showing epistaxis. E. Anal bleeding in adult rabbit.

Supplementary Table 1. Top 10 strains with highest nucleotide identity with the Algerian strains obtained by nucleotide Blast (nBlast) for the VP60+VP10 sequences.

Strain	Top-10 strains (nBlast)	Query Cover	E value	Identity (%)	Accession	Country	Date
**052**	Rabbit hemorrhagic disease virus isolate BC/Canada/WIN-AH-2018-OTH-0024	99%	0.0	97.68%	MT900570.1	Canada	Feb-18
	Rabbit hemorrhagic disease virus isolate BC/Canada/WIN-AH-2018-OTH-0032	99%	0.0	97.58%	MT900572.1	Canada	Mar-18
	Rabbit hemorrhagic disease virus isolate BC/Canada/WIN-AH-2018-OTH-0029	99%	0.0	97.58%	MT900571.1	Canada	Feb-18
	Rabbit hemorrhagic disease virus 2 strain RHDV2/Apr2020/AZ1	99%	0.0	97.54%	MT506237.2	Canada	Feb-18
	Rabbit hemorrhagic disease virus 2 strain RHDV2/Apr2020/TX1	99%	0.0	97.49%	MT506233.2	USA	Apr-20
	Rabbit hemorrhagic disease virus strain Senasica_20	99%	0.0	97.44%	OM973948.1	Mexico	Mar-20
	Rabbit hemorrhagic disease virus strain MexChihua2020	99%	0.0	97.44%	MT982431.1	Mexico	Apr-20
	Rabbit hemorrhagic disease virus 2 isolate S2008982	99%	0.0	97.39%	MW926377.1	USA	2020
	Rabbit hemorrhagic disease virus isolate BC/Canada/WIN-AH-2019-OTH-0022	99%	0.0	97.39%	MT900573.1	Canada	Mar-19
	Rabbit hemorrhagic disease virus 2 isolate D2014080	99%	0.0	97.34%	MW926372.1	USA	2020
**068**	Rabbit hemorrhagic disease virus isolate BC/Canada/WIN-AH-2018-OTH-0024	99%	0.0	97.54%	MT900570.1	Canada	Feb-18
	Rabbit hemorrhagic disease virus isolate BC/Canada/WIN-AH-2018-OTH-0032	99%	0.0	97.44%	MT900572.1	Canada	Mar-18
	Rabbit hemorrhagic disease virus isolate BC/Canada/WIN-AH-2018-OTH-0029	99%	0.0	97.44%	MT900571.1	Canada	Feb-18
	Rabbit hemorrhagic disease virus 2 strain RHDV2/Apr2020/AZ1	99%	0.0	97.39%	MT506237.2	Canada	Feb-18
	Rabbit hemorrhagic disease virus 2 strain RHDV2/Apr2020/TX1	99%	0.0	97.34%	MT506233.2	USA	Apr-20
	Rabbit hemorrhagic disease virus strain Senasica_20	99%	0.0	97.29%	OM973948.1	Mexico	Mar-20
	Rabbit hemorrhagic disease virus strain MexChihua2020	99%	0.0	97.29%	MT982431.1	Mexico	Apr-20
	Rabbit hemorrhagic disease virus 2 isolate S2008982	99%	0.0	97.25%	MW926377.1	USA	2020
	Rabbit hemorrhagic disease virus isolate BC/Canada/WIN-AH-2019-OTH-0022	99%	0.0	97.25%	MT900573.1	Canada	Mar-19
	Rabbit hemorrhagic disease virus 2 isolate D2014080	99%	0.0	97.20%	MW926372.1	USA	2020
**076**	Rabbit hemorrhagic disease virus isolate BC/Canada/WIN-AH-2018-OTH-0024	99%	0.0	97.68%	MT900570.1	Canada	Feb-18
	Rabbit hemorrhagic disease virus isolate BC/Canada/WIN-AH-2018-OTH-0032	99%	0.0	97.58%	MT900572.1	Canada	Mar-18
	Rabbit hemorrhagic disease virus isolate BC/Canada/WIN-AH-2018-OTH-0029	99%	0.0	97.58%	MT900571.1	Canada	Feb-18
	Rabbit hemorrhagic disease virus 2 strain RHDV2/Apr2020/AZ1	99%	0.0	97.54%	MT506237.2	Canada	Feb-18
	Rabbit hemorrhagic disease virus 2 strain RHDV2/Apr2020/TX1	99%	0.0	97.49%	MT506233.2	USA	Apr-20
	Rabbit hemorrhagic disease virus strain Senasica_20	99%	0.0	97.44%	OM973948.1	Mexico	Mar-20
	Rabbit hemorrhagic disease virus strain MexChihua2020	99%	0.0	97.44%	MT982431.1	Mexico	Apr-20
	Rabbit hemorrhagic disease virus 2 isolate S2008982	99%	0.0	97.39%	MW926377.1	USA	2020
	Rabbit hemorrhagic disease virus isolate BC/Canada/WIN-AH-2019-OTH-0022	99%	0.0	97.39%	MT900573.1	Canada	Mar-19
	Rabbit hemorrhagic disease virus 2 isolate S2100208	99%	0.0	97.29%	MW926380.1	USA	2021
**083**	Rabbit hemorrhagic disease virus isolate BC/Canada/WIN-AH-2018-OTH-0032	99%	0.0	97.63%	MT900572.1	Canada	Mar-18
	Rabbit hemorrhagic disease virus isolate BC/Canada/WIN-AH-2018-OTH-0029	99%	0.0	97.63%	MT900571.1	Canada	Feb-18
	Rabbit hemorrhagic disease virus isolate BC/Canada/WIN-AH-2018-OTH-0024	99%	0.0	97.63%	MT900570.1	Canada	Feb-18
	Rabbit hemorrhagic disease virus isolate BC/Canada/WIN-AH-2019-OTH-0022	99%	0.0	97.44%	MT900573.1	Canada	Mar-19
	Rabbit hemorrhagic disease virus 2 strain RHDV2/Apr2020/AZ1	99%	0.0	97.39%	MT506237.2	USA	Apr-20
	Rabbit hemorrhagic disease virus 2 strain RHDV2/Apr2020/TX1	99%	0.0	97.34%	MT506233.2	USA	Apr-20
	Rabbit hemorrhagic disease virus strain Senasica_20	99%	0.0	97.29%	OM973948.1	Mexico	Mar-20
	Rabbit hemorrhagic disease virus strain MexChihua2020	99%	0.0	97.29%	MT982431.1	Mexico	Apr-20
	Rabbit hemorrhagic disease virus 2 isolate S2008982	99%	0.0	97.25%	MW926377.1	USA	2020
	Rabbit hemorrhagic disease virus 2 isolate S2100208	99%	0.0	97.15%	MW926380.1	USA	2021
**027**	Rabbit hemorrhagic disease virus isolate BC/Canada/WIN-AH-2018-OTH-0024	100%	0.0	97.73%	MT900570.1	Canada	Feb-18
	Rabbit hemorrhagic disease virus isolate BC/Canada/WIN-AH-2018-OTH-0032	100%	0.0	97.63%	MT900572.1	Canada	Mar-18
	Rabbit hemorrhagic disease virus isolate BC/Canada/WIN-AH-2018-OTH-0029	100%	0.0	97.63%	MT900571.1	Canada	Feb-18

	Rabbit hemorrhagic disease virus 2 strain RHDV2/Apr2020/AZ1	100%	0.0	97.59%	MT506237.2	USA	Apr-20
	Rabbit hemorrhagic disease virus 2 strain RHDV2/Apr2020/TX1	100%	0.0	97.54%	MT506233.2	USA	Apr-20
	Rabbit hemorrhagic disease virus strain Senasica_20	100%	0.0	97.49%	OM973948.1	Mexico	Mar-20
	Rabbit hemorrhagic disease virus strain MexChihua2020	100%	0.0	97.49%	MT982431.1	Mexico	Apr-20
	Rabbit hemorrhagic disease virus 2 isolate S2100208	100%	0.0	97.34%	MW926380.1	USA	2021
	Rabbit hemorrhagic disease virus 2 isolate S2008982	100%	0.0	97.34%	MW926377.1	USA	2020
	Rabbit hemorrhagic disease virus 2 isolate D2100068	100%	0.0	97.34%	MW926374.1	USA	2021
**024**	Rabbit hemorrhagic disease virus isolate BC/Canada/WIN-AH-2018-OTH-0024	97%	0.0	98.25%	MT900570.1	Canada	Feb-18
	Rabbit hemorrhagic disease virus isolate BC/Canada/WIN-AH-2018-OTH-0032	97%	0.0	98.16%	MT900572.1	Canada	Mar-18
	Rabbit hemorrhagic disease virus isolate BC/Canada/WIN-AH-2018-OTH-0029	97%	0.0	98.16%	MT900571.1	Canada	Feb-18
	Rabbit hemorrhagic disease virus 2 strain RHDV2/Apr2020/AZ1	97%	0.0	97.98%	MT506237.2	USA	Apr-20
	Rabbit hemorrhagic disease virus 2 strain RHDV2/Apr2020/TX1	97%	0.0	97.89%	MT506233.2	USA	Apr-20
	Rabbit hemorrhagic disease virus isolate BC/Canada/WIN-AH-2019-OTH-0022	97%	0.0	97.89%	MT900573.1	Canada	Mar-19
	Rabbit hemorrhagic disease virus strain Senasica_20	97%	0.0	97.81%	OM973948.1	Mexico	Mar-20
	Rabbit hemorrhagic disease virus strain MexChihua2020	97%	0.0	97.81%	MT982431.1	Mexico	Apr-20
	Rabbit hemorrhagic disease virus 2 isolate S2008982	97%	0.0	97.72%	MW926377.1	USA	2020
	Rabbit hemorrhagic disease virus 2 isolate D2100068	97%	0.0	97.72%	MW926374.1	USA	2021
**002**	Rabbit hemorrhagic disease virus isolate Zar06-12	96%	0.0	97.63%	KP129399.1	Spain	2012
	Rabbit hemorrhagic disease virus isolate Jedaida_2/TUN/2019	96%	0.0	97.50%	MZ913391.1	Tunisia	2019
	Rabbit hemorrhagic disease virus isolate Jedaida_1/TUN/2019	96%	0.0	97.50%	MZ913390.1	Tunisia	2019
	Rabbit hemorrhagic disease virus isolate Touza_2/TUN/2019	96%	0.0	97.31%	MZ913395.1	Tunisia	2019
	Rabbit hemorrhagic disease virus isolate Touza_1/TUN/2019	96%	0.0	97.31%	MZ913394.1	Tunisia	2019
	Rabbit hemorrhagic disease virus isolate Rabbit_1512/TUN/2020	96%	0.0	97.31%	MZ913393.1	Tunisia	2020
	Rabbit hemorrhagic disease virus isolate Monastir_1/TUN/2018	96%	0.0	96.99%	MZ913392.1	Tunisia	2018
	Rabbit hemorrhagic disease virus strain RHDV/GER-BY/D35.L00714/2014	96%	0.0	96.73%	LR899192.1	Germany	2014
	Rabbit hemorrhagic disease virus isolate RHDV-N11	96%	0.0	96.60%	KM878681.1	Spain	2011
	Rabbit hemorrhagic disease virus isolate Tar06-12	96%	0.0	96.48%	KP129397.1	Spain	2012

Supplementary Table 2. Top 10 strains with highest nucleotide identity with the Algerian strains obtained by nucleotide Blast (nBlast) for the partial RdRp sequences.

Strain	Top-10 strains (nBlast)	Query Cover	E value	Identity (%)	Accession	Country	Date
**052**	Rabbit hemorrhagic disease virus 2 strain RHDV2/Apr2020/AZ1	100%	0.0	98.67%	MT506237.2	USA	Apr-20
	Rabbit hemorrhagic disease virus 2 strain RHDV2/Mar2020/NM1	100%	0.0	98.67%	MT506234.2	USA	Mar-20
	Rabbit hemorrhagic disease virus 2 strain RHDV2/Apr2020/TX1	100%	0.0	98.67%	MT506233.2	USA	Apr-20
	Rabbit hemorrhagic disease virus strain Senasica_20	100%	0.0	98.52%	OM973948.1	Mexico	Mar-20
	Rabbit hemorrhagic disease virus strain MexChihua2020	100%	0.0	98.52%	MT982431.1	Mexico	Apr-20
	Rabbit hemorrhagic disease virus 2 isolate S2101296	100%	0.0	98.52%	MW926381.1	USA	2021
	Rabbit hemorrhagic disease virus 2 isolate S2006894	100%	0.0	98.52%	MW926375.1	USA	2020
	Rabbit hemorrhagic disease virus 2 isolate D2100068	100%	0.0	98.52%	MW926374.1	USA	2021
	Rabbit hemorrhagic disease virus 2 isolate S2101766	100%	0.0	98.38%	MW926384.1	USA	2021
	Rabbit hemorrhagic disease virus 2 isolate T2002842	100%	0.0	98.38%	MW926383.1	USA	2020
**068**	Rabbit hemorrhagic disease virus 2 strain RHDV2/Apr2020/AZ1	100%	0.0	98.67%	MT506237.2	USA	Apr-20
	Rabbit hemorrhagic disease virus 2 strain RHDV2/Mar2020/NM1	100%	0.0	98.67%	MT506234.2	USA	Mar-20
	Rabbit hemorrhagic disease virus 2 strain RHDV2/Apr2020/TX1	100%	0.0	98.67%	MT506233.2	USA	Apr-20
	Rabbit hemorrhagic disease virus strain Senasica_20	100%	0.0	98.52%	OM973948.1	Mexico	Mar-20
	Rabbit hemorrhagic disease virus strain MexChihua2020	100%	0.0	98.52%	MT982431.1	Mexico	Apr-20
	Rabbit hemorrhagic disease virus 2 isolate S2101296	100%	0.0	98.52%	MW926381.1	USA	2021
	Rabbit hemorrhagic disease virus 2 isolate S2006894	100%	0.0	98.52%	MW926375.1	USA	2020
	Rabbit hemorrhagic disease virus 2 isolate D2100068	100%	0.0	98.52%	MW926374.1	USA	2021
	Rabbit hemorrhagic disease virus 2 isolate S2101766	100%	0.0	98.38%	MW926384.1	USA	2021
	Rabbit hemorrhagic disease virus 2 isolate T2002842	100%	0.0	98.38%	MW926383.1	USA	2020
**076**	Rabbit hemorrhagic disease virus 2 strain RHDV2/Apr2020/AZ1	100%	0.0	98.67%	MT506237.2	USA	Apr-20
	Rabbit hemorrhagic disease virus 2 strain RHDV2/Mar2020/NM1	100%	0.0	98.67%	MT506234.2	USA	Mar-20
	Rabbit hemorrhagic disease virus 2 strain RHDV2/Apr2020/TX1	100%	0.0	98.67%	MT506233.2	USA	Apr-20
	Rabbit hemorrhagic disease virus strain Senasica_20	100%	0.0	98.52%	OM973948.1	Mexico	Mar-20
	Rabbit hemorrhagic disease virus strain MexChihua2020	100%	0.0	98.52%	MT982431.1	Mexico	Apr-20
	Rabbit hemorrhagic disease virus 2 isolate S2101296	100%	0.0	98.52%	MW926381.1	USA	2021
	Rabbit hemorrhagic disease virus 2 isolate S2006894	100%	0.0	98.52%	MW926375.1	USA	2020
	Rabbit hemorrhagic disease virus 2 isolate D2100068	100%	0.0	98.52%	MW926374.1	USA	2021
	Rabbit hemorrhagic disease virus 2 isolate S2101766	100%	0.0	98.38%	MW926384.1	USA	2021
	Rabbit hemorrhagic disease virus 2 isolate T2002842	100%	0.0	98.38%	MW926383.1	USA	2020
**083**	Rabbit hemorrhagic disease virus 2 strain RHDV2/Apr2020/AZ1	100%	0.0	98.38%	MT506237.2	USA	Apr-20
	Rabbit hemorrhagic disease virus 2 strain RHDV2/Mar2020/NM1	100%	0.0	98.38%	MT506234.2	USA	Mar-20
	Rabbit hemorrhagic disease virus 2 strain RHDV2/Apr2020/TX1	100%	0.0	98.38%	MT506233.2	USA	Apr-20
	Rabbit hemorrhagic disease virus strain Senasica_20	100%	0.0	98.23%	OM973948.1	Mexico	Mar-20
	Rabbit hemorrhagic disease virus strain MexChihua2020	100%	0.0	98.23%	MT982431.1	Mexico	Apr-20
	Rabbit hemorrhagic disease virus 2 isolate S2101296	100%	0.0	98.23%	MW926381.1	USA	2021
	Rabbit hemorrhagic disease virus 2 isolate S2006894	100%	0.0	98.23%	MW926375.1	USA	2020
	Rabbit hemorrhagic disease virus 2 isolate D2100068	100%	0.0	98.23%	MW926374.1	USA	2021
	Rabbit hemorrhagic disease virus 2 isolate S2101766	100%	0.0	98.08%	MW926384.1	USA	2021
	Rabbit hemorrhagic disease virus 2 isolate T2002842	100%	0.0	98.08%	MW926383.1	USA	2020
**027**	Rabbit hemorrhagic disease virus 2 strain RHDV2/Apr2020/AZ1	100%	0.0	98.08%	MT506237.2	USA	Apr-20
	Rabbit hemorrhagic disease virus 2 strain RHDV2/Mar2020/NM1	100%	0.0	98.08%	MT506234.2	USA	Mar-20
	Rabbit hemorrhagic disease virus 2 strain RHDV2/Apr2020/TX1	100%	0.0	98.08%	MT506233.2	USA	Apr-20

	Rabbit hemorrhagic disease virus isolate BC/Canada/WIN-AH-2019-OTH-0022	100%	0.0	98.08%	MT900573.1	Canada	Mar-19
	Rabbit hemorrhagic disease virus isolate BC/Canada/WIN-AH-2018-OTH-0032	100%	0.0	98.08%	MT900572.1	Canada	Mar-18
	Rabbit hemorrhagic disease virus isolate BC/Canada/WIN-AH-2018-OTH-0029	100%	0.0	98.08%	MT900571.1	Canada	Feb-18
	Rabbit hemorrhagic disease virus isolate BC/Canada/WIN-AH-2018-OTH-0024	100%	0.0	98.08%	MT900570.1	Canada	Feb-18
	Rabbit hemorrhagic disease virus strain Senasica_20	100%	0.0	97.93%	OM973948.1	Mexico	Mar-20
	Rabbit hemorrhagic disease virus strain MexChihua2020	100%	0.0	97.93%	MT982431.1	Mexico	Apr-20
	Rabbit hemorrhagic disease virus 2 isolate S2101296	100%	0.0	97.93%	MW926381.1	USA	2021
**024**	Rabbit hemorrhagic disease virus 2 strain RHDV2/Apr2020/AZ1	100%	0.0	98.38%	MT506237.2	USA	Apr-20
	Rabbit hemorrhagic disease virus 2 strain RHDV2/Mar2020/NM1	100%	0.0	98.38%	MT506234.2	USA	Mar-20
	Rabbit hemorrhagic disease virus 2 strain RHDV2/Apr2020/TX1	100%	0.0	98.38%	MT506233.2	USA	Apr-20
	Rabbit hemorrhagic disease virus strain Senasica_20	100%	0.0	98.23%	OM973948.1	Mexico	Apr-20
	Rabbit hemorrhagic disease virus strain MexChihua2020	100%	0.0	98.23%	MT982431.1	Mexico	Apr-20
	Rabbit hemorrhagic disease virus 2 isolate S2101296	100%	0.0	98.23%	MW926381.1	USA	2021
	Rabbit hemorrhagic disease virus 2 isolate S2006894	100%	0.0	98.23%	MW926375.1	USA	2020
	Rabbit hemorrhagic disease virus 2 isolate D2100068	100%	0.0	98.23%	MW926374.1	USA	2021
	Rabbit hemorrhagic disease virus 2 isolate S2101766	100%	0.0	98.08%	MW926384.1	USA	2021
	Rabbit hemorrhagic disease virus 2 isolate T2002842	100%	0.0	98.08%	MW926383.1	USA	2020
**002**	Rabbit hemorrhagic disease virus isolate Zar06-12	63%	0.0	98.38%	KP129399.1	Spain	2012
	Rabbit hemorrhagic disease virus isolate Tar06-12	63%	0.0	98.15%	KP129397.1	Spain	2012
	Rabbit hemorrhagic disease virus strain RHDV/GER-NW/EI15-7.L03600/2016	63%	0.0	97.92%	LR899170.1	Germany	2016
	Rabbit hemorrhagic disease virus strain RHDV/GER-BY/D35.L00714/2014	63%	0.0	97.92%	LR899192.1	Germany	2014
	Rabbit hemorrhagic disease virus isolate Seg08-12	63%	0.0	97.69%	KP129396.1	Spain	2012
	Rabbit hemorrhagic disease virus isolate RHDV-N11	63%	0.0	97.69%	KM878681.1	Spain	2011
	Rabbit hemorrhagic disease virus isolate Rabbit_1512/TUN/2020	63%	0.0	97.46%	MZ913393.1	Tunisia	2020
	Rabbit hemorrhagic disease virus isolate Jedaida_2/TUN/2019	63%	0.0	97.46%	MZ913391.1	Tunisia	2019
	Rabbit hemorrhagic disease virus isolate Jedaida_1/TUN/2019	63%	0.0	97.46%	MZ913390.1	Tunisia	2019
	Rabbit hemorrhagic disease virus isolate CBVal16	63%	0.0	97.23%	KM979445.1	Portugal	2016
